# Evaluation of thermoregulation of horses (*Equus caballus*) submitted to two methods of post-exercise cooling, in hot and humid climate conditions, in the Eastern Amazon

**DOI:** 10.3389/fvets.2023.1150763

**Published:** 2023-04-17

**Authors:** Bruna Rafaela Ferreira Lisboa, Jamile Andréa Rodrigues da Silva, Welligton Conceição da Silva, Antônio Vinícius Corrêa Barbosa, Lilian Kátia Ximenes Silva, José de Brito Lourenço-Júnior

**Affiliations:** ^1^Institute of Animal Health and Production, Federal Rural University of the Amazon (UFRA), Belem, Pará, Brazil; ^2^Postgraduate Program in Animal Science (PPGCAN), Institute of Veterinary Medicine, Federal University of Para (UFPA), Federal Rural University of the Amazon (UFRA), Brazilian Agricultural Research Corporation (EMBRAPA), Castanhal, Pará, Brazil; ^3^Cyberspace Institute, Federal Rural University of the Amazon (UFRA), Belem, Pará, Brazil; ^4^Institute of Veterinary Medicine, Federal University of Pará (UFPA), Castanhal, Pará, Brazil

**Keywords:** weather, heat, horses, sport, equine

## Abstract

In Brazil, the study on equine thermoregulation has not shown considerable advances, moreover, in the Amazon, this is a little explored aspect. This study aims to evaluate the thermoregulation of horses submitted to two methods of post-exercise cooling, in the climatic conditions of the Eastern Amazon. The experiment was conducted at Centro Hipico, Ananindeua, Para, for 15 days. Ten male horses, castrated of the Brazilian breed, aged 13 years and with an average weight of 482.3 kg were used. Equestrianism, which was exercised with pre-established protocol in the arena and track, for 30 min. After the exercise, the animals were divided into two groups for application of the treatments, which were two cooling methods, such as a bath with water at room temperature (about 25°C) and a hiper cooling method (6–9°C). During the experimental period, air temperature (AT) and relative humidity (RH) data were recorded and the Temperature and Humidity Index (THI) was calculated. Data from the rectal temperature (RT), heart rate (HR), respiratory rate (RR) and Body surface temperature (BST) of the animals were also measured at three moments: before exercise, after exercise and after applying the cooling methods. The adaptability index used was the Benezra Thermal Comfort Index (BTCI). The BST was performed with the help of infrared thermograph, which were the left side of the neck, thorax, rump, and right side to the armpi, before the exercise, after the exercise and after applying the cooling methods. The statistical design was completely randomized. Analysis of variance was performed using the GLM Procedure of SAS 9.1.3. After the application of the cooling techniques in the animals, the AT and THI were higher and the highest RH values were found before the exercise (87.21%). The highest RT, RR, HR, BST, and BTCI values were observed after exercise. There was no significant dierence (*P* > 0.05) between cooling methods. There was a highly significant and positive correlation (*P* < 0.01) between all physiological variables (RT, RR, HR, and BST) and the Iberian Index with AT and THI and negative with RH (*P* < 0.01), thus demonstrating the influence of the environment on the thermoregulation of animals. It is concluded the evaluation of stress, welfare and thermoregulation of horses submitted to two methods of post-exercise cooling, in the climatic conditions of the Eastern Amazon, demonstrated that the studied cooling methods showed to reduce the rectal temperature, the respiratory rate, the heart rate and the body surface temperature with equal efficiency. However, in terms of practical ease of use, the room temperature water cooling method has proven to be more practical.

## 1. Introduction

Environmental changes affect thermoregulatory performance, particularly in high-demanding animals such as horses. These two sentences are general statements that will have at least one or more references to where they were taken from. Equine farming has become an important part of Brazilian agribusiness, moving the economy in Brazil. According to the Brazilian Institute of Geography and Statistics, it is estimated that Brazil has 5,962,126 heads of horses, with the state of Pará being the fifth state to have the highest number of horses, around 458,145 head. Of these horses, ~3,790 head are located in the municipality of Santarém, western Pará (1). It is valid to consider that the interaction of animals with the environment must be considered when seeking efficiency in animal performance, as the different responses of the animal to the peculiarities of each region are decisive in the success of the productive activity (2).

The control of heat exchange between body surface and external environment plays a very important role in regulation of body temperature during different physiological phases and/or activities throughout life of homeotherms. Thermoregulatory adjustments can be induced not only by changes in environmental temperature but also by a variety of physiological situations including age, fasting and food intake, stress circumstances and inflammation status inducing changes in internal temperature which are followed by changes in body surface temperature (3).

In the Amazon, temperature and relative humidity are high, as well as rainfall and solar radiation. These climatic variables, when associated with inadequate management of animals and pastures, can be considered stressful elements and negatively reflect on animal performance, in addition to preventing their productive and reproductive potential in cattle (4–7), sheep and goats (8), buffaloes (6) and horses (9–11).

In order to assess the ability of animals to adjust to the prevailing environmental conditions in regions with hot climates, there are methods that are classified as “adaptability measures,” as they allow verifying the ability of animals to maintain their homeothermy or dissipate heat (12). Such methods are based on the physiological variables of the animals, such as rectal temperature, respiratory rate and body surface temperature (13, 14). The evaluation of surface temperature through infrared thermography, a non-invasive technique that allows obtaining a distance image of a specific body region, represents a valuable tool to monitor the physiologic status, welfare and the stress responses of domestic animals (15, 16). The Benezra Comfort Index stands out in the literature, used to quantify the thermal stress in animals at a given time and place (17, 18).

Horses used for sport are even more tolerant to thermal stress (19, 20), especially when exposed to environments with high temperatures and relative humidity of the air (21), because in this situation thermogenesis is aggravated and health risks occur exponential exponentially form. This fact has been worrying trainers, who must adopt forms of cooling so that the animal returns to thermal comfort (22). In the literature, it is possible to evidence different applications of tested cold methods to refresh sport sports horses after exercise (20, 23–27).

In Brazil, the study on equine thermoregulation has not shown considerable advances, moreover, in the Amazon, this is a little explored aspect. This study presents the hypothesis that post-exercise cooling in horses can be achieved through the application of an ice water bath or hyper cooling (HRC). Based on this information, this study aims to evaluate the thermoregulation of horses submitted to two methods of post-exercise cooling, in the climatic conditions of the Eastern Amazon.

## 2. Material and methods

### 2.1. Ethics committee

The experiment was approved by the Ethics Committee, protocol No. 054/2015 (CEUA) and 23084.013102/2015-01 (UFRA).

### 2.2. Location

This research was carried out at Centro Hípico (latitude 1°23′33.4″ south and longitude 48°24′27.6″ west), Ananindeua, Pará. The climate is tropical rainforest, tropical humid in all seasons (Af) Köppen, characterized by not having a defined dry period, with a rainy season, from December to May, and a less rainy season, from June to November. The average annual temperature of 26.7°C, relative humidity 84%, rainfall of 3,001 mm and 2,338 h of sunlight (4).

### 2.3. Experimental animals

Ten castrated male horses of the Brasileiro de Hipismo (BH) breed, ~13 years old and average weight of 482.3 kg. As an inclusion criterion, clinically healthy horses with uniform weight were used, used for sport were used. The feed diet consisted of feed and pasture, with Tifton (*Cynodon* spp.) and *Brachiaria brizantha* cv. Marandu, which were cut and offered in the trough at 11:00 am and 5:00 pm. The ration was pelleted, Equiplus brand, and was provided at 6:00 am, 2:00 pm and 8:00 pm. Drinking water was provided *ad libitum*.

The exercise protocol was designed with the aim of increasing body temperature without loss of fluids through sweating, so as not to compromise responses to cooling (28). The horses were saddled and mounted and then exercised in a riding arena for about 30 min. After the exercise, the animals were divided into two groups for the application of the experimental treatments, which are two cooling methods, such as a bath with ice water or hyper cooling (HRC) and other bath with natural water at room temperature (AN). HRC consists of pouring water at a temperature of 6 to 9°C over the animal's back, letting it remain for about a minute, so that there is heat exchange with the body surface, and removing the excess with the help of a squeegee, when a new layer of cold water is poured over the coat, repeating the procedure. The AN method was obtained through the same procedure as the HRC, differing only in the temperature of the water, which was obtained from the tap. These methods were applied immediately after the exercise and the readings of the physiological variables, 10 min after the application of the methods.

### 2.4. Rectal temperature and respiratory rate and heart rate

The regulatory parameters of rectal temperature (RT), respiratory rate (RR) and heart rate (HR) were evaluated in three moments: before exercise, after exercise and after cooling methods. To obtain the RT, a veterinary clinical thermometer (Model-5198.10, Incoterm R, São Paulo, Brazil) was used, with a scale of up to 44°C, with the reading result expressed in degrees centigrade. The RR was observed by checking and counting the thoracoabdominal movements, during for 1 min. The HR was measured with the help of a veterinary stethoscope, where the heart hugs were counted for 1 min.

### 2.5. Body surface temperature

The BST was performed with the help of an ITTI 380 infrared thermometer (Instrutemp^®^, São Paulo, Brazil) operated at a maximum distance of one meter from the measurement points on the animal, which were the left side of the neck, thorax, rump, and right side to the armpit, having obtained the average of these values, in three moments: before the exercise, after the exercise and after applying the cooling methods.

### 2.6. Meteorological data

Meteorological data, air temperature (AT) and relative humidity (RH) were recorded using a data logger, model AKSO AK172, installed at the experimental site. The readings of the environmental variables were performed at the time of the physiological data collection measurements. From these climatic variables, the Temperature and Humidity Index was calculated.

### 2.7. Temperature and humidity index

The THI was calculated at from Equation 1 (29).


$$\begin{array}{l} \text{THI}=\left(\text{1}.\text{8}\times {\text{T}}^{{}^{\circ}}+\text{32}\right)-\left(0.\text{55}-0.\text{55}\times \text{HR}/\text{1}00\right)\\ \;\times \left(\text{1}.\text{8}\times {\text{T}}^{{}^{\circ}}-\text{26}\right) \end{array}$$

T° = Air temperature expressed in °C.

HR = Relative humidity expressed in %.

In this study, THI was considered up to 70 indicate a non-stressful environment, among 71 and 78 critical, between 79 and 83 dangerous and above 83, emergency condition (9).

### 2.8. Benezra thermal comfort index

The assessment of the animals' adaptability to the environment was analyzed by applying two indices. Benezra Thermal Comfort Index (BTCI) (30), was obtained through the formula:


$$\begin{array}{l} \text{BTCI}=\text{RT}/\text{38}+\text{RR}/\text{16} \end{array}$$

where RT = Rectal temperature (°C) and RR = Respiratory rate.

This test has been adapted for horses, where 38 is the average normal equine rectal temperature (31) and 16 is the average normal equine respiratory rate (32). For horses, the closer the BTCI index is to value 2, the more adapted the animal will be to heat or a challenging environment. To record all physiological and climatic variables, data collection forms were used in three moments of equine activities: Before Exercise, After Exercise and After Cooling.

### 2.9. Statistical analysis

The statistical design was completely randomized. Analysis of variance was performed using the GLM Procedure of SAS 9.1.3 (33) to verify the effect of cooling methods on physiological parameters and adaptability index. The Shapiro-Wilk normality test was applied, showing normal data. Means were compared using the Tukey Kramer test, at 5% probability or *p* < 0.05, and Pearson's linear correlations were performed between physiological and climatic variables.

## 3. Results

Of all the environmental factors and climate is the most influential on animal performance. The average values of the climatic variables, during the experimental period, are presented in Table 1.

**Table 1:** Means and standard error of the variables and climate index were measured at three evaluation times for horses: before exercise, after exercise and after cooling down, in the experimental area, in Ananindeua, Pará.

**Variables**	**Before exercise**	**After exercise**	**After cooling**
Air temperature (°C)	26.71 ± 0.10^c^	28.48 ± 0.16^b^	29.13 ± 0.20^a^
Relative humidity (%)	87.21 ± 0.27^a^	82.70 ± 0.44^b^	80.93 ± 0.50^c^
Temperature and humidity index (THI)	78.48 ± 0.15^c^	80.77 ± 0.21^b^	81.54 ± 0.25^a^

Average ab followed by distinct lower case letters, in the same column, are different (p < 0.05).

**Figure 1:**
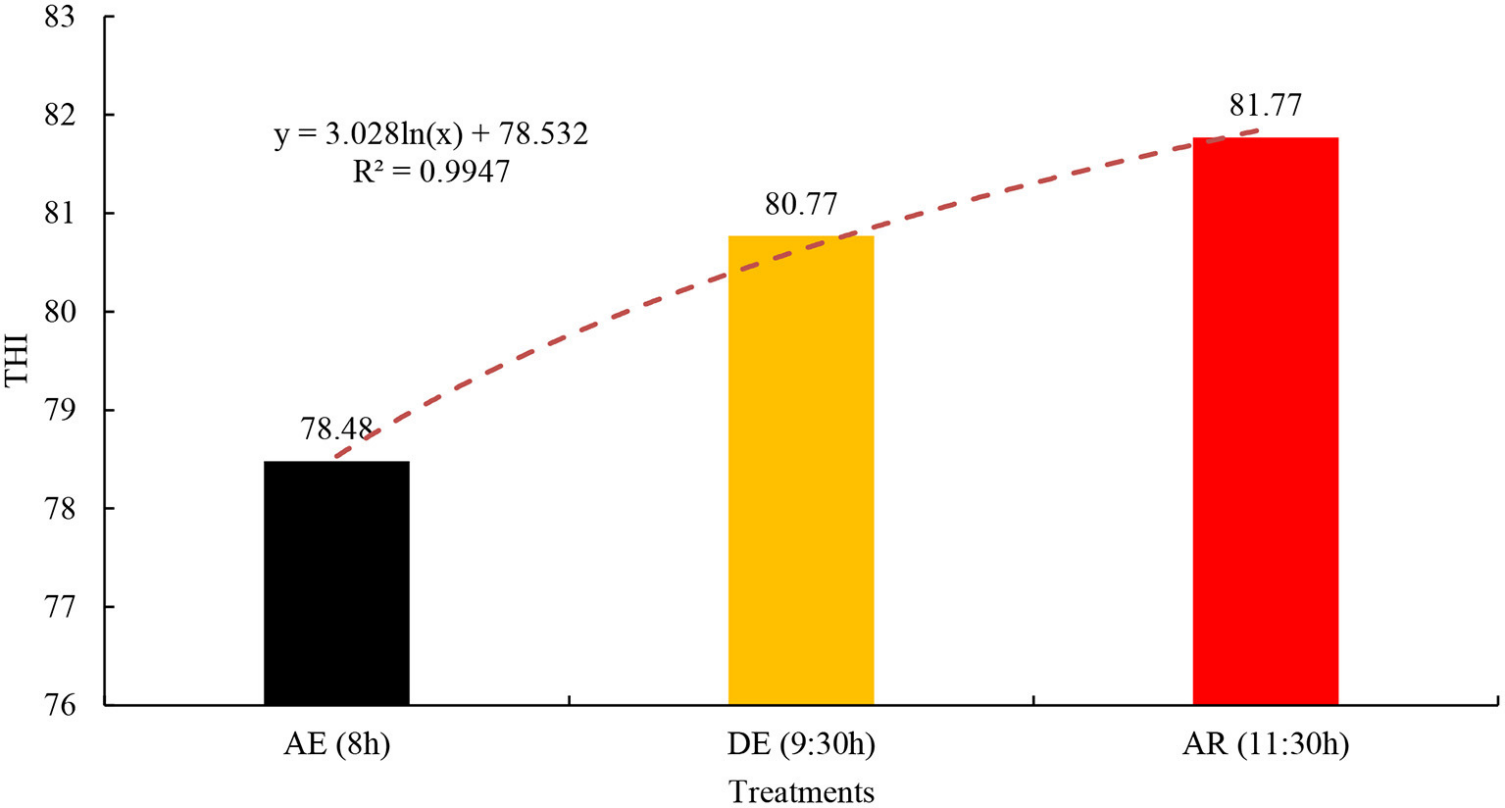
Variation of the average values of the temperature and humidity index, in three moments of equine activities, in Ananindeua, Pará, Brazil. AE, before exercise; DE, after exercise; AR, after cooling.

There was a significant difference between the moments of activity of the horses (*p* < 0.05) for the AT variable, where after the application of the cooling techniques in the animals, AT was higher. This fact is due to the time at which this procedure was performed, as it occurred at the end of the morning, around 11:30 am (Figure 1). In this study, the THI indicated that there was thermal stress, reaching values between 71 and 78 critical on this scale and between 79 and 83 dangerous (9).

The climatic variables demonstrated that the climate can cause thermal discomfort to the animals, but it is necessary to evaluate the physiological reactions of the horses to confirm their state of comfort and adaptability to the environment. The averages of the physiological variables and adaptability indexes of the experimental animals of the three moments of the experiment, such as, before the exercise, after the exercise and after the cooling (Table 2).

**Table 2:** Means and standard deviation of physiological variables and adaptability indices of horses, before exercise, after exercise and after cooling down, in the experimental area, in Ananindeua, Pará.

**Variables**	**Before exercise**	**After exercise**	**After cooling**
Rectal temperature (°C)	37.63 ± 0.04^c^	39.03 ± 0.06^a^	38.71 ± 0.05^b^
Respiratory rate (mov./min)	24.48 ± 0.45^c^	69.12 ± 1.84^a^	43.44 ± 1.67^b^
Heart rate (beats/min)	34.24 ± 0.64^c^	54.66 ± 0.99^a^	42.54 ± 0.87^b^
Skin temperature (°C)	31.46 ± 0.15^c^	34.6 ± 0.12^a^	33.39 ± 0.11^b^

Average ab followed by distinct lower case letters, in the same column, are different (p < 0.05).

The RT is the result of the difference between the thermal energy produced plus that received by the animal organism and the thermal energy dissipated from them to the environment. There was a significant difference in the RT between the three moments of equine activity (*p* < 0.05), where the highest average value was observed after exercise.

Horses exposed to the sun, and performing physical activity, rapidly increase the RT, above 40°C. In the present research, it was observed that the RR was also higher after the exercise (*p* < 0.05). The HR, likewise, was higher (*p* < 0.05) after the exercise of the experimental animals. BST was also higher after exercise (*p* < 0.05). The Benezra Index was significantly higher (*p* < 0.05) after exercise. Before exercise, the average value was 2.17, close to 2.0, considered ideal, which suggests that during this time (about 8 h), the animals are more adaptable, even after cooling down, around 11 am. h, when the values reached 3.19. These results can possibly be explained by the low AT and high RH, which occur during the night, and minimize the need for thermolysis by the animals in the early morning.

It is important to highlight that after the application of the cooling methods, the animals started to recover the values of the physiological variables, however, not with a high speed, probably due to the climate, which was already adverse, with high values of AT (29, 13°C) and THI (81.54).

There was no significant difference (*p* > 0.05) between the cooling methods (Table 3), which indicates that both methods are efficient to restore the physiological parameters of horses to their normal values. The mean values observed were far from 2.0, considered inadequate, suggesting that the animals were within a challenging stress zone.

**Table 3:** Means and standard deviation of physiological variables and adaptability index of horses submitted to two cooling methods, in Ananindeua, Pará.

**Variables**	**Natural water**	**Hyper cooling**
Rectal temperature (°C)	38.76 ± 0.07	38.67 ± 0.06
Respiratory rate (mov./min)	42.91 ± 2.5	43.97 ± 2.24
Heart rate (beats/min)	44.58 ± 1.52	40.56 ± 0.72
Skin temperature (°C)	33.48 ± 0.19	33.29 ± 0.11
BCTI	3.16 ± 0.13	3.22 ± 0.11

BCTI, Benezra thermal comfort index.

The correlations between physiological variables and adaptability and climate indices are shown in Table 4.

**Table 4:** Correlation between the physiological variables of horses and the adaptability index with climate variables in Ananindeua, Pará.

**Parameters**	**AT**	**RH**	**THI**
RT (°C)	0.413^†^^†^	−0.386^†^^†^	0.416^†^^†^
RR (mov./min)	0.348^†^^†^	−0.342^†^^†^	0.349^†^^†^
HR (beats/min)	0.286^†^^†^	−0.244^†^^†^	0.297^†^^†^
BST (°C)	0.607^†^^†^	−0.597^†^^†^	0.603^†^^†^
Benezra index	0.353^†^^†^	0.351^†^^†^	0.352^†^^†^

†† Significant at 1%. RT, rectal temperature; RR, respiratory rate; HR, heart rate; BST, body surface temperature; THI, temperature and humidity index; AT, air temperature; RH, relative air humidity.

There was a highly significant and positive correlation (*p* < 0.01) between all physiological variables (RT, RR, HR, and BST) and the Index of Iberia with AT and THI and negative with RH (*p* < 0.01), thus demonstrating the influence of the environment on the thermoregulation of the animals.

## 4. Discussion

During the day there are moments that are some moments are more or less favorable to the thermal comfort of the animals, and this process is mediated by the radiation balance, that is, by the accounting between the receipt and return of radiation, which is very variable throughout the day and year, which promotes daily and annual changes in air temperature (34, 35).

Likewise, the analysis of variance detected a significant difference in the relative humidity between the moments of activities, where the highest values were found before the exercise (87.21%). These data show that the average daily course of relative humidity is inverse to that of air temperature. It is important to remember that the thermal comfort of the animals depends to a high degree on the levels of relative humidity in the air, in association with the air temperature (36).

Temperature and Humidity Index values up to 70 indicate a non-stressful environment, between 71 and 78, critical, between 79 and 83 dangerous and above 83, an emergency condition (9). In the present research, there was a significant difference in the temperature and Humidity Index between the moments of activity of the horses. These results indicate that breeders should avoid animals being exposed to the sun and performing physical activities at times of higher air temperature, as thermogenesis can be accentuated, and these factors lead to cerebral hyperthermia (22).

The rectal temperature is the result of the difference between the thermal energy produced plus that received by the animal organism and the thermal energy dissipated from them to the environment. There was a significant difference in the rectal temperature between the three moments of equine activity, where the highest average value was observed after exercise, surpassing the variation range for equines, which is 37.2–38.2°C (37). Before the exercise, the animals were in thermoneutrality and during the exercise there was an increase in rectal temperature.

Horses exposed to the sun, and performing physical activity, rapidly increase the rectal temperature, above 40°C. If the rectal temperature exceeds 42°C, it indicates the need for immediate refrigeration (12). Hyperthermia of this magnitude can also signal the need for a reduction in the intensity or duration of exercise in a hot environment (22).

In exercising horses, metabolic rates increase 10–20 times (38). Only 20% of the energy obtained through biochemical processes of transformation of the energy substrates used during the exercise are transformed into effective physical work, the remaining 80% are transformed into heat, which is accumulated in the animal's body, initially, with the aim of improving muscle biochemical reactions. This accumulation of heat can cause less efficiency in the performance of physical activities, therefore, the thermoregulation of the animal must be efficient, in order to lose heat to the environment (39, 40). In addition, when the horse cannot thermoregulate efficiently, the manifestation of exertional heat illness may occur, which is linked to inadequate physiological responses (41, 42).

In the present research, it was observed that the respiratory rate was also higher after the exercise. This occurred because the increase in respiratory rate is one of the thermoregulation strategies of horses. Many mammals use the thermoregulatory system to lose heat through evaporative cooling, and to do so, they breathe more through their mouths than through their noses. Horses, on the other hand, due to their anatomical characteristics, cannot breathe through their mouths, however, the vascularization of the nasal turbulence, the nasal mucosa and the bronchial tree indicate that the respiratory role is important for thermoregulation during exercise and in its recovery (43, 44).

Even in mammals that do not pant, such as horses, evaporative heat loss from the respiratory tract is likely to increase during prolonged exercise because dead space ventilation increases (45). Dead space is found within the alveoli, which originates from alveoli with poor blood perfusion (alveolar dead space), and from regions of the nasal cavity, pharynx, larynx, trachea, bronchi, and bronchioles (anatomical dead space). The dead space has this denomination, due to the fact that gas exchanges do not occur ideally in the region. Horses under heat stress will increase respiratory rate and dead space ventilation in an attempt to lose heat (46). In hot, humid climates, when cutaneous evaporative cooling is compromised, respiratory heat loss may account for a relatively larger proportion of total heat loss and therefore account for 25% or more of total heat loss (22).

The heart rate, likewise, was higher after the exercise of the experimental animals. When horses are under heat stress or during exercise, the demand for blood increases due to the increased need for irrigation of muscle mass and the diversion of blood to the skin to dissipate heat. To meet these demands, the horse's body reacts by increasing the heart rate, thus increasing the amount of blood pumped per minute and consequently there is an increase in blood pressure at appropriate levels. In addition, the increase in sweating, which occurs due to physical activities in a hot environment, results in dehydration, with a reduction in blood volume, also reducing systolic volume, therefore, to maintain cardiac output and tissue perfusion, it is heart rate increased (47, 48).

Body surface temperature was also higher after exercise. This result occurs because when the horse activates its thermoregulatory system, there is an increase in blood flow from the central region of the body to the periphery of the body, causing peripheral vasodilation in an attempt to eliminate heat, which results in an increase in body surface temperature (49).

These results indicate that there is no advantage in using the hyper cooling technique instead of using natural water at room temperature, since for the application of this method of hyper cooling it is necessary to have costs for its preparation. Janczarek et al. (20) showed that cooling methods using water caused a decrease in rectal and body surface temperature.

As a limitation, it was not possible to adopt infrared thermography, considered a tool capable of evaluating specific regions with vasomotor alterations mediated by the nervous system. However, the laser the thermograph used in this research makes it possible to evaluate the surface temperature efficiently, and its use is recommended (13, 50), as it is a tool with greater sensitivity, specificity and analysis capacity when compared to the infrared thermometer, this being a field of action that should be widely studied in the future.

## 5. Conclusions

The evaluation of thermoregulation of horses submitted to two methods of post-exercise cooling, in the climatic conditions of the Eastern Amazon, demonstrated that the studied cooling methods showed to reduce the rectal temperature, the respiratory rate, the heart rate and the body surface temperature with equal efficiency. However, in terms of practical ease of use, the room temperature water cooling method has proven to be more practical. In addition, it was noted that there is an influence of climate on the physiological and adaptability parameters of horses in the Eastern Amazon, therefore, it is recommended to avoid handling animals for exercise at times with higher temperatures.

## Data availability statement

The original contributions presented in the study are included in the article/supplementary material, further inquiries can be directed to the corresponding author.

## Ethics statement

The experiment was approved by the Ethics Committee, protocol No. 054/2015 (CEUA) and 23084.013102/2015-01 (UFRA).

## Author contributions

Experiment design: BL, JS, and JL-J. Experiment perform: WS, AB, LS, BL, JS, and JL-J. Data curation: BL, JS, AB, WS, and JL-J. Formal analysis: AB and WS. Writing–original draft: JS, JL-J, AB, and WS. All authors edited and approved the final manuscript.
